# Switch to mania after ayahuasca consumption in a man with bipolar disorder: a case report

**DOI:** 10.1186/s40345-014-0020-y

**Published:** 2015-02-24

**Authors:** Alejandro G Szmulewicz, Marina P Valerio, Jose M Smith

**Affiliations:** Hospital de Emergencias Psiquiatricas Torcuato de Alvear, Av Warnes 2630, Capital Federal, Argentina; Bipolar Disorder Program, Neurosciences Institute, Favaloro University, Buenos Aires, Argentina

**Keywords:** Ayahuasca, Bipolar disorder, DSM 5, Switch to mania, MAO inhibitors

## Abstract

**Background:**

There is an increasing use of ayahuasca for recreational purposes. Furthermore, there is a growing evidence for the antidepressant properties of its components. However, there are no reports on the effects of this substance in the psychiatric setting. Harmaline, one of the main components of ayahuasca, is a selective and reversible MAO-A inhibitor and a serotonin reuptake inhibitor.

**Case report:**

We present the case of a man with bipolar disorder who had a manic episode after an ayahuasca consumption ritual. This patient had had at least one hypomanic episode in the past and is currently depressed. We discuss the diagnostic repercussion of this manic episode.

**Conclusion:**

There is lack of specificity in the diagnosis of substance-induced mental disorder. The knowledge of the pharmacodynamic properties of ayahuasca consumption allows a more physiopathological approach to the diagnosis of the patient.

## Background

Ayahuasca is a psychotropic, hallucinogenic beverage, composed of a mixture of Amazonian plants. It is usually consumed as an infusion with *Banisteriopsis caapi* and *Psychotria viridis* (Guimaraes dos Santos 2010). The main components of *B. caapi* are β-carbolines - harmine and tetrahydroharmine - and to a lesser extent harmaline, harmol, and harmalol, while *P. viridis* contains a tryptamine, *N*,*N*-dimethyltryptamine (DMT), and a 5HT 2A agonist (Guimaraes dos Santos 2010; Callaway et al. 2005; McKenna et al. 1998). DMT, from *P. viridis* species, is the psychoactive substance of ayahuasca (Guimaraes dos Santos 2010). DMT is orally inactive due to intestinal MAO-A metabolism. Harmine, harmaline, and tetrahydroharmine are reversible, competitive, selective inhibitors of MAO-A (Guimaraes dos Santos 2010), with almost no effect on MAO-B (Herraiz 2010). When DMT is orally administered, it is peripherally inactivated by MAO-A (Riba et al. 2012). Nevertheless, when it is combined with a peripheral MAO-A inhibitor (like harmine), its oral biodisponibility increases, this interaction being the base of ayahuasca psychotropic effect (McKenna 2004).

## Case presentation

We present the case of a 30-year-old Argentinian man, single and currently unemployed, who lives with his mother. He arrived at our emergency room in order to continue treatment after being discharged from a hospital in Brazil. It was in there where he was admitted due to an acute psychotic episode after being involved in a 4-day ritual of ayahuasca consumption.

The patient had traveled to Brazil 3 months before in order to learn about South American tribes. In this context, he was offered to take part in an ayahuasca consumption ritual. Two days after the last consumption, he began having mystical and paranoid delusional ideas, auditory hallucinations, racing thoughts, disorganized behavior, elevated energy, and euphoria. The psychotic symptoms were consistent with his euphoric mood. He was taken to a psychiatric hospital where he received risperidone 2 mg/day and clonazepam 2 mg/day. After a month of being admitted, he was asymptomatic and was discharged with the same medication and traveled back to Argentina to continue his treatment. According to his mother (and by the patient retrospectively), the day before the ayahuasca ritual, the patient did not present any of the manic symptoms previously described. He maintained a coherent speech and slept 8 h per day.

The patient is the youngest brother in a family of middle socioeconomic status. He had a eutocic birth and reached developmental milestones at appropriate times. When he was 6 years old, he was enrolled in primary school where he developed asthma, enuresis, and night terrors. He was a sociable child and reports no substance abuse disorder. He graduated from high school and started a course of business management. His family background revealed that his father suffered bipolar disorder type I.

Two weeks before the journey to Brazil, the patient presented a period of increased energy and goal-directed activity, sleep disorder, pressured speech, increased self-esteem, and running thoughts that lasted for 10 days, compatible with a hypomanic episode. He states that he had experienced this kind of episodes several times in the past. There was no previous history of manic or depressive episodes.

As per an actual mental state examination in our hospital, the amount and speed of speech were diminished, and his affect was depressed. His thought content presented ideas of hopelessness and ruin. He had no delusions or hallucinations. He showed marked anhedonia, apathy, and clinophilia. His sleep and appetite were normal. This depressive episode started immediately after the remission of the psychotic episode described above.

### Discussion

We have presented a case of a patient with a previous history of hypomanic episodes and a first-degree family history of bipolar disorder. In the context of ayahuasca consumption, this patient developed a manic episode with psychotic features, which leads us to wonder about its etiology.

Substance-induced mental disorders are diagnosed according to DSM 5 when the characteristic symptoms of the disorder appear during or up to 1 month after the consumption of a substance, if there is no data to justify a primary disorder (i.e., symptoms preceding the use of the substance) (American Psychiatric Association 2013).

After conducting a review, it is seen that in most cases, the clinical features of ayahuasca-induced psychotic disorder are similar: sensory perception disturbances (the most frequent visual hallucinations), elevated body temperature, cardiovascular events (bradycardia, hypotension), psychomotor agitation, ataxia, tremors, and vomiting (Frison et al. 2008; Mahmoudian et al. 2002; Ben Salah et al. 1986).

The presence of preexisting hypomanic episodes, the presence of a positive family history of bipolar disorder, the absence of associated neurovegetative symptoms, and the fact that ayahuasca psychotic episodes are rare and transient during consumption do not support a diagnosis of either psychotic or bipolar disorder induced by ayahuasca nor ayahuasca intoxication. In addition, a revision made by Gable and colleagues (Gable 2007) reports that psychotic episodes induced by ayahuasca components are rare (under 1%) and resolve spontaneously in a few hours.

For some time, data postulating the antidepressant action of one component of ayahuasca, harmine, began to appear. This component may have inhibitory actions on serotonin reuptake and agonism of the serotonin 1A receptor. It also acts as a reversible inhibitor of MAO-A enzyme (Guimaraes dos Santos 2010; Ng et al. 2008; Glennon et al. 2000; Herraiz et al. 2010).

In summary, we believe that this patient had an antidepressant-induced mania due to excessively prolonged use of a substance with antidepressant properties and his bipolar disorder. There are studies that suggest a 4.58% (Tondo et al. 2010) to 15% (Pickar 1982) risk of switch to mania in bipolar patients treated with MAO inhibitors.

According to DSM 5, ‘a manic episode that emerges during antidepressant treatment but persists beyond the physiological effect of treatment is sufficient evidence of manic episode and therefore a bipolar disorder type I’ (American Psychiatric Association 2013); given that for high doses of β-carbolines the half-life is 1 to 5 h (Riba et al. 2003), we believe this is the case of the patient presented.

## Conclusions

In our patient, manic symptoms were caused by the antidepressant effect of the substance and by his bipolar disorder diagnosis. The reported case illustrates a lack of specificity in the diagnosis of a substance-induced psychotic episode and suggests a decreased threshold to manic episodes in bipolar patients by any compound with antidepressant action. Additionally, it emphasizes the utility of DSM 5 modification of the antidepressant-induced manic symptoms as exclusion criteria for a bipolar disorder diagnosis.

Finally, this case describes another mechanism of toxicity for this substance in bipolar patients and accentuates the need for caution in the psychiatric setting.

## Consent

Written informed consent was obtained from the patient for the publication of this case report. A copy of the written consent is available for review by the Editor-in-Chief of this journal.
